# The progressive journey of poor-responder neovascular AMD: tracking structural evolution and visual decline over time

**DOI:** 10.1038/s41433-026-04306-6

**Published:** 2026-02-17

**Authors:** Ilaria Lolli, Maria Grazia Pignataro, Alba Chiara Termite, Giulia Ribezzi, Giacomo Boscia, Enrico Borrelli, Michele Reibaldi, Giovanni Alessio, Francesco Boscia, Pasquale Viggiano

**Affiliations:** 1 Department of Translational Biomedicine Neuroscience, University of Bari “Aldo Moro”, Bari, Italy; 2 Department of Surgical Sciences, University of Turin, Turin, Italy

**Keywords:** Predictive markers, Retinal diseases

## Abstract

**Purpose:**

To characterise the natural history of poor-responder neovascular age-related macular degeneration (AMD) by tracking structural evolution and visual decline over time.

**Methods:**

This retrospective longitudinal study analysed 70 eyes of 70 treatment-naive neovascular AMD patients who completed loading dose therapy, received ≥7 injections in the first year, and experienced ≥10 ETDRS letter visual acuity (BCVA) loss from post-loading baseline. Spectral-domain OCT imaging and BCVA were evaluated at three timepoints: baseline (post-loading), 10-letter loss, and worst visual outcome. Multivariate regression analysis identified independent predictors of visual acuity at each timepoint. Primary structural parameters assessed included macular atrophy, subretinal fibrosis, external limiting membrane (ELM) and ellipsoid zone (EZ) integrity, central retinal thickness (CRT), and fluid parameters.

**Results:**

Mean follow-up was 38.5 ± 22.8 months. Macular atrophy progression was dramatic (7.1% → 41.4% → 81.4%, *p* < 0.001) and subretinal fibrosis increased substantially (11.4% → 25.7% → 57.1%, *p* < 0.001). CRT showed paradoxical biphasic evolution (262.6 → 278.6 → 252.0 μm). Multivariate analysis revealed three distinct phases: no independent predictors at baseline, comprehensive multi-pathway model at 10-letter loss with subretinal fibrosis (*β* = −0.536), hyperreflective material (*β* = −0.350), and intraretinal fluid (*β* = −0.223) as independent predictors (R² = 0.428), and fibrotic dominance at worst outcome where subretinal fibrosis emerged as the sole predictor (*β* = −0.469, R² = 0.220). CRT showed no predictive value across all timepoints.

**Conclusions:**

Poor-responder neovascular AMD follows a three-phase evolutionary journey with subretinal fibrosis as the dominant independent predictor of visual decline. These findings demand a paradigmatic shift toward qualitative structural assessment focusing on fibrotic changes rather than thickness-based monitoring.

## Introduction

Anti-VEGF therapy has revolutionised the management of neovascular age-related macular degeneration (AMD), with central retinal thickness (CRT) becoming the cornerstone parameter for monitoring treatment response and disease activity [1, 2]. The widespread adoption of CRT-based protocols stems from its objective, quantifiable nature and strong initial correlations with visual outcomes in landmark clinical trials [3]. Current standard practise equates CRT increase with disease recurrence, guiding retreatment decisions and follow-up intervals across ophthalmology practises worldwide [4].

However, a growing body of evidence questions the universal applicability of CRT-centric monitoring, particularly in patients demonstrating suboptimal treatment responses [5]. Despite achieving apparent anatomical control with stable or reduced CRT measurements, a significant proportion of patients—estimated at 20–30%—experience progressive visual deterioration during long-term anti-VEGF therapy [6, 7]. This phenomenon of “poor responders” represents a critical gap in our understanding of treatment failure mechanisms and challenges the fundamental assumptions underlying current monitoring paradigms [8].

The observed dissociation between morphometric stability and functional decline indicates that CRT alone may not adequately reflect the multifaceted nature of retinal degeneration in neovascular AMD [7]. Recent investigations have highlighted the importance of qualitative OCT parameters, including outer retinal integrity, macular atrophy development, and subretinal fibrosis, in predicting long-term visual outcomes [9–13]. These structural changes may represent more fundamental pathological processes that escape detection through conventional thickness measurements.

Macular atrophy, particularly within the central 3 mm zone critical for central vision, has emerged as a critical determinant of visual prognosis in AMD, representing irreversible photoreceptor and RPE loss [14, 15]. Similarly, subretinal fibrosis development signals the transition from an inflammatory to a cicatricial disease phase, with profound implications for visual recovery potential [16, 17]. Outer retinal integrity, including external limiting membrane (ELM) and ellipsoid zone (EZ) continuity, has emerged as a critical determinant of visual recovery potential [18, 19]. Despite their recognised clinical importance, the relative contribution of these structural changes compared to CRT alterations in driving visual deterioration remains incompletely characterised.

The clinical implications of this knowledge gap are substantial. Patients with stable CRT measurements may receive false reassurance about disease control, while potentially reversible structural changes go undetected and untreated. Furthermore, the timing and sequence of these degenerative processes may offer insights into optimal intervention windows and personalised treatment strategies.

To address these critical questions, we conducted a comprehensive retrospective longitudinal analysis of poor-responder neovascular AMD patients, tracking the evolution of structural parameters alongside visual function.

## Methods

### Study design and population

This retrospective longitudinal study was conducted within the medical retina research unit of the Department of Translational Biomedicine and Neuroscience at the University of Bari Aldo Moro, Italy. The research methodology adhered strictly to the ethical principles outlined in the Declaration of Helsinki. As per Italian regulations for retrospective studies, formal approval from an ethics committee was not required, but the institutional ethics committee was informed and provided written acknowledgment.

**Fig. 1:**
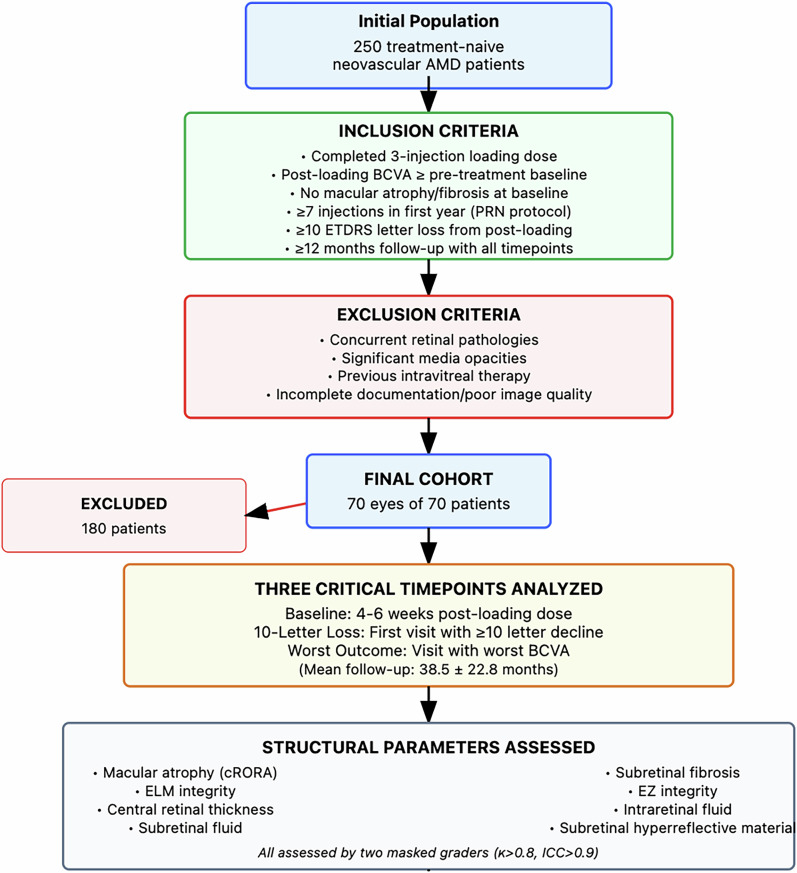
Flowchart depicting the systematic patient selection process for the poor-responder neovascular AMD cohort. Starting from 250 treatment-naive patients, strict inclusion and exclusion criteria were applied to identify 70 patients who experienced ≥10 ETDRS letter visual acuity loss despite ongoing anti-VEGF therapy. All patients completed three critical timepoints: baseline (4–6 weeks post-loading dose), 10-letter loss (first documentation of ≥10 letter decline), and worst visual outcome (visit with poorest BCVA during follow-up). Structural parameters were assessed using spectral-domain OCT by two masked graders with high inter-rater agreement (*κ* > 0.8 for categorical variables, ICC > 0.9 for continuous measures).

We initially identified 250 patients with treatment-naive neovascular AMD from our electronic medical records. After applying strict inclusion and exclusion criteria (i.e., see below and Fig. 1), 70 eyes of 70 patients comprised the final study cohort.

#### Inclusion criteria

Treatment-naive neovascular AMD patients who completed a standard 3-injection loading dose regimen with post-loading BCVA equal to or superior to pre-treatment baseline, had no evidence of macular atrophy or subretinal fibrosis at treatment initiation, received intravitreal anti-VEGF therapy (ranibizumab 0.5 mg, aflibercept 2 mg, or bevacizumab 1.25 mg) following a 3-injection loading dose regimen with subsequent pro re nata (PRN) retreatment based on disease activity [20], experienced ≥10 ETDRS letter BCVA loss from post-loading baseline, and had minimum 12-month follow-up encompassing all three critical timepoints (baseline, 10-letter loss, and worst visual outcome), with high-quality spectral-domain OCT imaging available at each timepoint. Treatment switches between agents were permitted as part of routine clinical management.

#### Exclusion criteria

Concurrent retinal pathologies affecting central vision, significant media opacities preventing adequate OCT acquisition, previous intravitreal therapy history, incomplete clinical documentation, or inadequate image quality precluding reliable structural assessment [21].

### OCT analysis protocol

Three critical timepoints were retrospectively analysed:

- Baseline: structural OCT 4–6 weeks post-loading dose (optimal anatomical response)
- 10-letter loss: First visit documenting ≥10 letter BCVA decline from baseline
- Worst visual outcome: first visit with worst BCVA during follow-up

In patients where the worst visual outcome coincided with the 10-letter loss timepoint (i.e., no further deterioration occurred beyond the initial 10-letter decline), the worst visual outcome timepoint was defined as the subsequent follow-up visit after the 10-letter loss documentation to ensure temporal distinction between the two analytical timepoints.

All examinations were performed using Heidelberg Spectralis spectral-domain OCT (Heidelberg Engineering, Germany). Spectral-domain OCT volumetric scans (20 × 20°, 49 B-scans, 16 ART mean) were centred on the fovea. A minimum signal strength of 25 was required to the OCT images to be included, as recommended by the manufacturer [22]. Best-corrected visual acuity was measured in ETDRS letters using standardised charts. Two masked (PV and EB), experienced graders independently assessed structural parameters using established criteria according to current international guidelines with high inter-rater agreement (κ > 0.8 for categorical variables, ICC > 0.9 for continuous measures).

### Structural parameter assessment

#### Primary structural parameters

- Macular atrophy: Well-demarcated areas of complete outer retinal and RPE atrophy (cRORA) within central 3 mm, characterised by photoreceptor and RPE loss with choroidal hypertransmission [11, 14].
- Subretinal fibrosis: Organised hyperreflective subretinal material with architectural characteristics indicating cicatricial tissue formation [16]. Fibrosis diagnosis required longitudinal confirmation (≥3 months stability) to distinguish organised cicatricial tissue from acute hyperreflective materials including haemorrhage, fibrin, and acute SHRM.
- External limiting membrane (ELM) integrity: Continuity assessment within central 1 mm using ETDRS grid mapping [23].
- Ellipsoid zone (EZ) integrity: Presence and continuity of inner/outer segment junction within central 1 mm [24].
- Central retinal thickness: Automated measurement within central 1 mm ETDRS subfield [25].
- Fluid parameters: Presence and distribution of intraretinal fluid (hyporeflective spaces within neurosensory retina) and subretinal fluid (hyporeflective space between neurosensory retina and RPE), assessed as important indicators of disease activity and treatment response [23].

### Statistical analysis

Descriptive statistics included means, standard deviations, and ranges for continuous variables, and frequencies with percentages for categorical variables. Temporal evolution of structural parameters was assessed using McNemar test for paired categorical data to evaluate changes in binary morphological features (presence/absence of atrophy, fibrosis, ELM integrity, EZ integrity, and fluid parameters) between timepoints. For continuous variables such as CRT, paired t-tests were used to compare means between consecutive timepoints. Statistical significance for temporal changes was assessed by comparing baseline values to subsequent timepoints (10-letter loss and worst visual outcome) using appropriate paired tests. Structure-function relationships were analysed using multivariate linear regression analysis to identify independent predictors of visual acuity at each timepoint. All structural parameters were included as potential predictors in the initial model, using a stepwise approach with forward selection based on statistical significance. Multicollinearity was assessed using variance inflation factors (VIF), with values > 5 indicating concerning collinearity. Model fit was evaluated using R² values, and assumptions of normality and homoscedasticity were verified through residual analysis. All analyses were performed using SPSS version 28.0 (IBM Corporation, Armonk, NY, USA) with significance set at *p* < 0.05.

## Results

### Patient characteristics

Seventy eyes of 70 patients met inclusion criteria after screening 250 initial candidates. The study population demonstrated typical characteristics of neovascular AMD patients with advanced disease. Mean patient age was 76.8 ± 8.2 years (range: 58–89 years), with a female predominance (64.3%, 45/70). Cardiovascular risk factors were prevalent, with hypertension affecting 68.6% (48/70) of patients and diabetes mellitus present in 24.3% (17/70). Smoking history was documented in 41.4% (29/70) of patients, including 18.6% (13/70) current smokers and 22.8% (16/70) former smokers.

Baseline ophthalmologic examination revealed mean BCVA of 70.5 ± 12.8 letters (range: 37–85). MNV subtypes distribution showed type 1 predominance with 38.6% (27/70), followed by type 2 in 27.1% (19/70), type 3 in 20.0% (14/70), and polypoidal choroidal vasculopathy in 14.3% (10/70). Mean baseline (i.e., after the loading dose) CRT measured 262.6 ± 66.0 μm.

The temporal characteristics of disease progression showed considerable heterogeneity. Time to 10-letter loss varied dramatically from 8 to 70 months (mean: 23.7 ± 15.4 months), highlighting the unpredictable progression patterns that characterise poor-responder phenotypes. Mean follow-up to worst visual outcome was 38.5 ± 22.8 months (range: 12–85 months), demonstrating the chronic nature of deterioration in this population (Table 1).

**Table 1:** Baseline characteristics of study population (*n* = 70).

Characteristic	Value
**Demographics**	
Age, years (mean ± SD)	76.8 ± 8.2
Female sex, n (%)	45 (64.3)
**Systemic Comorbidities**	
Hypertension, n (%)	48 (68.6)
Diabetes mellitus, n (%)	17 (24.3)
Smoking history, n (%)	29 (41.4)
- Current smokers	13 (18.6)
- Former smokers	16 (22.8)
**Ophthalmologic Characteristics**	
BCVA at baseline, letters (mean ± SD)	70.5 ± 12.8
BCVA range, letters	37–85
Central retinal thickness, μm (mean ± SD)	262.6 ± 66.0
**MNV Subtype Distribution**	
Type 1, n (%)	27 (38.6)
Type 2, n (%)	19 (27.1)
Type 3, n (%)	14 (20.0)
PCV, n (%)	10 (14.3)

### Temporal evolution of structural parameters

#### Macular atrophy development

**Fig. 2:**
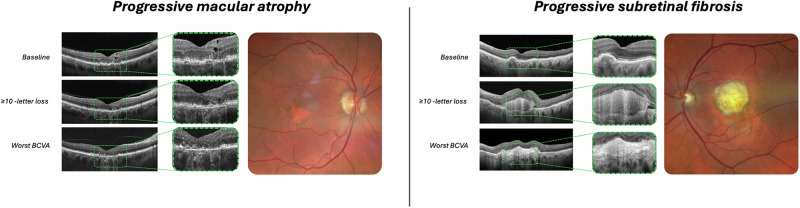
Representative cases demonstrating distinct patterns of progressive structural deterioration in poor responders to anti-VEGF therapy. Spectral-domain OCT B-scans through the fovea (wide field with magnified insets of the foveal region) and corresponding colour fundus photography at worst visual outcome for two representative cases. (Left) Progressive macular atrophy characterised by complete outer retinal loss with pronounced choroidal hypertransmission at worst visual outcome, corresponding to atrophic area visible on fundus examination. (Right) Progressive subretinal fibrosis characterised by organised hyperreflective subretinal material with posterior shadowing at worst visual outcome, corresponding to dense fibrotic scar visible on fundus examination. Both cases demonstrate the structural endpoints associated with irreversible visual deterioration despite continued anti-VEGF treatment. Dashed boxes indicate magnified regions.

Macular atrophy showed relentless progression across all timepoints, emerging as the dominant structural change. At baseline (i.e., after the loading dose), only 5/70 eyes (7.1%) presented with established macular atrophy. This increased dramatically to 29/70 eyes (41.4%) at the 10-letter loss timepoint (*p* < 0.01), representing 24 new cases of atrophy development. By worst visual outcome, 57/70 eyes (81.4%) demonstrated macular atrophy (*p* < 0.001 vs 10-letter timepoint), with an additional 28 eyes developing this irreversible change during the later disease course. Overall, 52/70 patients (74.3%) developed new macular atrophy during the study period (*p* < 0.001 baseline vs worst) (Table 2 and Fig. 2).

**Table 2:** Temporal evolution of structural parameters.

Parameter	Baseline	10-Letter Loss	Worst Outcome	p-value^a^
Macular Atrophy, n (%)	5 (7.1)	29 (41.4)†	57 (81.4)‡	<0.001
Subretinal Fibrosis, n (%)	8 (11.4)	18 (25.7)†	40 (57.1)‡	<0.001
ELM Integrity, n (%)	32 (45.7)	16 (22.9)†	9 (12.9)‡	<0.001
EZ Integrity, n (%)	30 (42.9)	19 (27.1)†	7 (10.0)‡	<0.001
Central Retinal Thickness, μm (mean ± SD)	262.6 ± 65.6	278.6 ± 116.2	252.0 ± 88.8	0.173
Intraretinal Fluid, n (%)	26 (37.1)	26 (37.1)	27 (38.6)	0.856
Subretinal Fluid, n (%)	31 (44.3)	27 (38.6)	17 (24.3)‡	<0.001

^a^Overall comparison across all timepoints using McNemar test for categorical variables and paired t-test for continuous variables †p < 0.05 vs baseline ‡p < 0.05 vs 10-letter loss timepoint.

*ELM* external limiting membrane, *EZ* ellipsoid zone, *SD* standard deviation.

#### Subretinal fibrosis evolution

Fibrotic changes demonstrated a different temporal pattern with continuous progression throughout follow-up. Baseline fibrosis (i.e., after the loading dose) was present in 8/70 eyes (11.4%). At 10-letter loss, this increased substantially to 18/70 eyes (25.7%) (*p* < 0.001), indicating 10 new cases of fibrosis development. By worst visual outcome, further significant progression occurred with 40/70 eyes (57.1%) showing fibrosis (*p* < 0.001 vs 10-letter timepoint), representing an additional 22 cases. Overall, 32/70 patients (45.7%) developed new subretinal fibrosis during follow-up (*p* < 0.001 baseline vs worst) (Table 2 and Fig. 2).

#### Outer retinal integrity changes

ELM integrity within central 1 mm showed marked deterioration from baseline (i.e., after the loading dose) 32/70 eyes (45.7%) to 16/70 eyes (22.9%) at 10-letter loss (*p* < 0.001), with further decline to 9/70 eyes (12.9%) at worst visual outcome (*p* = 0.041). Similarly, EZ integrity within central 1 mm declined from 30/70 eyes (42.9%) at baseline (i.e., after the loading dose) to 19/70 eyes (27.1%) at 10-letter loss (*p* = 0.002), and ultimately to 7/70 eyes (10.0%) at worst visual outcome (*p* = 0.003) (Table 2).

#### Central retinal thickness evolution

CRT demonstrated a biphasic pattern that contradicted conventional expectations. Mean thickness increased from 262.6 ± 65.6 μm at baseline (i.e., after the loading dose) to 278.6 ± 116.2 μm at 10-letter loss (*p* = 0.118), then unexpectedly decreased to 252.0 ± 88.8 μm at worst visual outcome (*p* = 0.173) (Table 2).

### Fluid parameter dynamics

Intraretinal fluid showed not significant improvement: 26/70 eyes (37.1%) at baseline, 26/70 eyes (37.1%) at 10-letter loss, and 27/70 eyes (38.6%) at worst visual outcome. Likewise, subretinal fluid demonstrated more pronounced resolution: 31/70 eyes (44.3%) at baseline, 27/70 eyes (38.6%) at 10-letter loss (*p* = 0.512), and 17/70 eyes (24.3%) at worst visual outcome (*p* < 0.001) (Table 2).

### Structure-function relationship analysis

Multivariate linear regression analysis revealed dynamic, stage-dependent relationships between structural parameters and visual function, with distinct predictive models emerging across the disease progression timepoints.

*Baseline multivariate analysis* failed to identify any structural parameters as independent predictors of visual acuity (all p > 0.10), indicating limited structure-function relationships in the early post-loading phase despite apparent morphometric control.

*At the 10-letter loss timepoint*, multivariate analysis identified three independent predictors of visual acuity in the final model (R² = 0.428, *p* < 0.001): subretinal fibrosis demonstrated the strongest predictive value (*β* = −0.536, *p* < 0.001), followed by subretinal hyperreflective material (*β* = −0.350, *p* = 0.001), and intraretinal fluid (*β* = −0.223, *p* = 0.025). No significant multicollinearity was detected among predictors (all VIF < 2.0). Notably, central retinal thickness, macular atrophy, and outer retinal integrity markers (ELM and EZ) failed to achieve independent significance in the multivariate model (all *p* > 0.10), despite their recognised clinical importance (Table 3).

**Table 3:** Multivariate linear regression analysis - all variables at 10-letter loss timepoint.

Variable	B	Standard Error	β (Standardised)	t	p-value
Subretinal Fibrosis	−17.031	3.145	−0.536	−5.415	**<0.001**
Subretinal Hyperreflective Material	−12.500	3.528	−0.350	−3.543	**0.001**
Intraretinal Fluid	−6.434	2.798	−0.223	−2.300	**0.025**
Central Retinal Thickness	−0.037	0.107	−0.043	−0.344	0.732
Subretinal Fluid	3.200	1.942	0.202	1.649	0.104
ELM Integrity	−0.148	1.961	−0.009	−0.075	0.940
EZ Integrity	1.550	2.066	0.094	0.752	0.455
Macular Atrophy (cRORA)	−2.633	1.603	−0.201	−1.642	0.106

Stepwise multiple linear regression analysis with forward selection (entry criterion *p* < 0.05, removal criterion *p* > 0.10). Dependent variable: best-corrected visual acuity (BCVA) in ETDRS letters. Model statistics: R² = 0.428, Adjusted R² = 0.402, *F* = 16.477, *p* < 0.001. Multicollinearity assessment: all variance inflation factors (VIF) < 2.0, indicating no significant collinearity among predictors. B unstandardised regression coefficient, β standardised regression coefficient, t t-statistic for testing H₀: *β* = 0, ELM external limiting membrane, EZ ellipsoid zone, ETDRS Early Treatment Diabetic Retinopathy Study, VIF variance inflation factor.

*At worst visual outcome*, multivariate analysis revealed subretinal fibrosis as the sole independent predictor of visual acuity (*β* = −0.469, *p* < 0.001, R² = 0.220). All other structural parameters, including macular atrophy and central retinal thickness, failed to contribute additional predictive value in the presence of fibrotic changes (all *p* > 0.10), suggesting that fibrosis dominates the structure-function relationship in advanced disease stages (Table S1).

*Temporal evolution of predictive models* revealed three distinct phases: Phase 1 (baseline) with no significant independent predictors; Phase 2 (10-letter loss) with emergence of a comprehensive multivariate model incorporating fibrotic, inflammatory, and fluid-related pathways; and Phase 3 (worst outcome) with simplification to fibrotic dominance as the sole remaining independent predictor.

## Discussion

Our longitudinal analysis of poor-responder neovascular AMD patients reveals a progressive degenerative cascade characterised by three distinct phases of structural deterioration. We found that 81.4% of patients developed macular atrophy and 57.1% developed subretinal fibrosis by worst visual outcome, with these irreversible changes occurring despite ongoing anti-VEGF therapy. Notably, our fibrosis prevalence aligns closely with the 10-year reported by Romano et al. [26]. in their comprehensive longitudinal study of neovascular AMD patients, supporting the reliability and generalisability of our findings across different populations and follow-up periods. Most significantly, our multivariate regression analysis identified subretinal fibrosis as the dominant independent predictor of visual decline, emerging at the 10-letter loss timepoint and remaining the sole significant predictor at worst outcome.

Our findings provide systematic validation for the qualitative OCT parameters. While CRT remains the primary quantitative endpoint in most clinical trials and protocols, practicing clinicians routinely assess structural integrity, fluid characteristics, and tissue organisation when making treatment decisions. By quantifying the predictive value of these qualitative parameters, we tried to establish an evidence-based hierarchy that supports clinical intuition with statistical rigour, enabling incorporation into standardised protocols and potentially redefining treatment endpoints beyond simple thickness measurements.

The multivariate models reveal the evolving pathophysiology underlying poor treatment response. At baseline, no structural parameters independently predicted visual outcomes, reflecting the apparent morphometric control achieved through initial anti-VEGF loading therapy. However, at the critical 10-letter loss timepoint, a comprehensive model emerged incorporating three independent predictors: subretinal fibrosis, subretinal hyperreflective material, and intraretinal fluid. This multi-pathway activation suggests that poor responders enter a phase of accelerated degeneration where inflammatory, vascular, and fibrotic processes converge to drive visual decline. The inclusion of intraretinal fluid as an independent predictor challenges traditional views of fluid as merely a treatment target, instead revealing its role as a marker of underlying neurodegeneration and predictor of poor visual outcomes [7].

By worst visual outcome, the predictive landscape simplified dramatically, with subretinal fibrosis emerging as the sole independent predictor. This transition from multi-factorial to fibrosis-dominated pathophysiology reflects the end-stage cicatricial transformation that characterises advanced poor-responder disease.

The counterintuitive finding that macular atrophy failed to achieve independent significance in multivariate models despite its high prevalence and established clinical importance deserves particular attention [8, 27]. This suggests that while atrophy represents irreversible photoreceptor loss, its functional impact may be mediated through or overshadowed by concurrent fibrotic processes. Alternatively, the binary nature of atrophy assessment may lack the granularity to capture functionally relevant variations in atrophic severity or topographic distribution that quantitative approaches might reveal [28].

Central retinal thickness demonstrated consistent lack of independent predictive value across all timepoints, reinforcing the fundamental inadequacy of thickness-based monitoring in poor-responder populations [6]. The paradoxical CRT reduction observed in our cohort likely reflects progressive tissue loss rather than therapeutic success, representing neurodegeneration masquerading as morphometric improvement [8, 29]. This phenomenon exposes critical limitations in conventional monitoring paradigms that equate thickness reduction with disease control.

Our findings establish intraretinal fluid as a significant risk factor for visual deterioration in poor responders, supporting emerging literature on fluid’s role in neurodegeneration [30]. Unlike the traditional view of fluid as simply indicative of vascular leak requiring anti-VEGF intensification, our data suggest that persistent intraretinal fluid in the setting of apparent morphometric control may signal ongoing neuroinflammatory processes that contribute independently to functional decline. This reframes fluid from a treatment target to a prognostic biomarker, with implications for risk stratification and treatment modification strategies.

The temporal sequence of our multivariate models reveals distinct therapeutic windows that current monitoring strategies may fail to recognise. The 10-letter loss timepoint emerges as a critical juncture where multiple pathological pathways activate simultaneously, potentially representing the last opportunity for intervention before irreversible fibrotic dominance ensues. Once widespread fibrosis develops, therapeutic options become severely limited, emphasising the importance of early structural parameter monitoring and aggressive intervention during the multi-pathway activation phase [8, 19, 31].

These findings demand a fundamental paradigm shift from quantitative thickness-based monitoring toward comprehensive qualitative structural assessment. Rather than relying primarily on CRT measurements, clinicians must systematically evaluate fibrotic changes, hyperreflective material accumulation, and fluid characteristics as primary markers of disease progression. The integration of artificial intelligence and advanced imaging modalities may facilitate this transition by enabling automated detection and quantification of these qualitative parameters.

The high prevalence of irreversible structural changes in our poor-responder cohort occurred despite adherence to current standard-of-care protocols, indicating that conventional anti-VEGF therapy alone may be insufficient to prevent progressive neurodegeneration in susceptible patients. This suggests the need for combination therapeutic strategies that address both vascular and neurodegenerative components of disease progression [29, 32].

Our cohort was treated with PRN protocols involving monthly monitoring during the first year with subsequent extension to 6–8 week intervals based on stability. The relatively early fibrosis development we observed raises the important question of whether treatment regimen selection influences structural outcomes. Disease activity fluctuations inherent to PRN approaches may permit inflammatory processes that drive tissue organisation and cicatrisation during monitoring intervals. More sustained disease control through treat-and-extend protocols or emerging longer-acting agents with extended durability might theoretically reduce fibrosis development by minimising these fluctuations.

Several limitations of our study design and methodology must be acknowledged when interpreting these findings and planning future investigations. Our 28% poor-responder rate exceeds typical reports (10-15%) as we captured ≥10-letter loss from post-loading baseline over extended follow-up, identifying progressive deterioration often missed in shorter studies. This broader definition, while clinically relevant, may limit direct comparisons with studies using stricter criteria. Moreover, the retrospective nature of our analysis inherently limits causal inference regarding the relationship between structural changes and functional decline. Additionally, our single-centre design may limit generalisability to diverse patient populations, treatment protocols, and imaging platforms used across different clinical settings. The absence of a control group of good-responder patients represents a significant limitation, preventing direct comparison of structural evolution patterns between poor and adequate treatment responders. The definition of poor-responder patients based on ≥10 letter vision loss may not capture the full spectrum of suboptimal treatment responses, potentially missing patients with subclinical progression or alternative deterioration patterns. Moreover, despite adherence to PRN protocols, may have influenced outcomes, as injection frequency, timing of retreatment decisions, and potential switches between anti-VEGF agents were not standardised across all patients. Additionally, our analysis focused on spectral-domain OCT parameters without incorporating newer imaging modalities such as OCT angiography, adaptive optics, or artificial intelligence-enhanced image analysis that may provide additional insights into disease progression mechanisms.

Despite these limitations, our analysis provides crucial insights into the natural history of poor-responder neovascular AMD. The identification of subretinal fibrosis as the dominant independent predictor of visual decline, coupled with the multi-phase evolution of pathophysiological processes, establishes a foundation for developing targeted interventions and personalised monitoring strategies. The transition from thickness-based to fibrosis-focused assessment represents not merely a technical refinement but a fundamental reconceptualisation of disease monitoring in this vulnerable patient population.

In conclusion, our analysis reveals the complex natural history of poor-responder neovascular AMD, characterised by progressive structural deterioration that follows independent pathways from functional decline. The journey of these patients progresses from a critical multi-pathway activation phase where subretinal fibrosis, SHRM, and IRF independently drive functional deterioration, to a final fibrosis-dominated phase where cicatricial changes become the sole determinant of visual outcomes, occurring despite apparent morphometric control through conventional CRT monitoring. The transition from quantity-based to quality-based structural assessment represents not merely a technical refinement but a paradigmatic shift essential for improving outcomes in this vulnerable patient population.

## Summary

### What was known before

- Poor-responder neovascular AMD patients experience progressive visual deterioration despite ongoing anti-VEGF therapy, affecting 20–30% of patients Central retinal thickness has been the cornerstone parameter for monitoring treatment response, but shows dissociation from functional outcomes in poor responders Qualitative OCT parameters including macular atrophy, subretinal fibrosis, and outer retinal integrity have been recognised as important prognostic factors The temporal sequence and relative contribution of structural changes to visual decline in poor responders remained incompletely characterised

### What this study adds

- Multivariate analysis reveals subretinal fibrosis as the dominant independent predictor of visual decline across all disease phases in poor responders Identifies three distinct evolutionary phases: no structural predictors at baseline, multi-pathway activation at 10-letter loss (fibrosis, hyperreflective material, intraretinal fluid), and fibrotic dominance at worst outcome Demonstrates that intraretinal fluid functions as an independent risk factor for visual deterioration, not merely a treatment target Shows that central retinal thickness lacks predictive value across all timepoints, reinforcing the inadequacy of thickness-based monitoring in poor responders Establishes the 10-letter loss timepoint as a critical therapeutic window where multiple pathological pathways remain potentially modifiable before irreversible fibrotic dominance

## Supplementary information

Table S1

## Data Availability

The datasets generated and analysed during the current study are not publicly available due to privacy concerns and regulations regarding patient data protection, but de-identified data are available from the corresponding author upon reasonable request and with appropriate institutional review board approval.
