# Supplementary material for: The progressive journey of poor-responder neovascular AMD: tracking structural evolution and visual decline over time
Source: Eye (Lond). 2026 Feb 17;40(6):827–34. doi: 10.1038/s41433-026-04306-6 (PMC13062072; doi:10.1038/s41433-026-04306-6)
Supplement: Supplementary file 1 — Table S1 [file 41433_2026_4306_MOESM1_ESM.docx]

| **Table S1.** Multivariate Linear Regression Analysis - All Variables at Worst Visual Outcome Timepoint | | | | | |
| --- | --- | --- | --- | --- | --- |
| **Variable** | **B** | **Standard Error** | **β (Standardized)** | **t** | **p-value** |
| **Subretinal Fibrosis** | **-17.125** | **3.912** | **-0.469** | **-4.378** | **<0.001** |
| Central Retinal Thickness | 0.014 | 0.019 | 0.081 | 0.750 | 0.456 |
| Intraretinal Fluid | 1.578 | 3.237 | 0.053 | 0.488 | 0.627 |
| Subretinal Fluid | 1.093 | 3.255 | 0.036 | 0.336 | 0.738 |
| ELM Integrity | -3.781 | 3.226 | -0.127 | -1.173 | 0.245 |
| EZ Integrity | 0.703 | 3.140 | 0.024 | 0.224 | 0.823 |
| Macular Atrophy | -0.922 | 3.257 | -0.031 | -0.283 | 0.778 |
| Subretinal Hyperreflective Material | 2.525 | 3.443 | 0.085 | 0.733 | 0.466 |
| Stepwise multiple linear regression analysis with forward selection (entry criterion p<0.05, removal criterion p>0.10). Dependent variable: best-corrected visual acuity (BCVA) in ETDRS letters. Model statistics: R² = 0.220, Adjusted R² = 0.208, F = 19.165, p < 0.001. Multicollinearity assessment: all variance inflation factors (VIF) < 2.0, indicating no significant collinearity among predictors. B = unstandardized regression coefficient; β = standardized regression coefficient; t = t-statistic for testing H₀: β = 0. **Abbreviations:** ELM = external limiting membrane; EZ = ellipsoid zone; ETDRS = Early Treatment Diabetic Retinopathy Study; VIF = variance inflation factor. | | | | | |
